# Gene flow between wild trees and cultivated varieties shapes the genetic structure of sweet chestnut (*Castanea sativa* Mill.) populations

**DOI:** 10.1038/s41598-022-17635-9

**Published:** 2022-09-02

**Authors:** Katarina Tumpa, Zlatko Šatović, Zlatko Liber, Antonio Vidaković, Marilena Idžojtić, Marin Ježić, Mirna Ćurković-Perica, Igor Poljak

**Affiliations:** 1 Department of Forestry, Institute of Forest Genetics, Dendrology and Botany, Faculty of Forestry and Wood Technology, University of Zagreb, 10000 Zagreb, Croatia; 2 Department for Seed Science and Technology, Faculty of Agriculture, University of Zagreb, 10000 Zagreb, Croatia; 3 Centre of Excellence for Biodiversity and Molecular Plant Breeding, 10000 Zagreb, Croatia; 4 Department of Biology, Faculty of Science, University of Zagreb, 10000 Zagreb, Croatia

**Keywords:** Plant sciences, Plant domestication, Population genetics

## Abstract

Gene flow between cultivated and wild gene pools is common in the contact zone between agricultural lands and natural habitats and can be used to study the development of adaptations and selection of novel varieties. This is likely the case in the northern Adriatic region, where centuries-old cultivated orchards of sweet chestnut (*Castanea sativa* Mill.) are planted within the natural distribution area of the species. Thus, we investigated the population structure of several orchards of sweet chestnuts. Furthermore, the genetic background of three toponymous clonal varieties was explored. Six genomic simple sequence repeat (gSSR) and nine EST-derived SSR (EST-SSR) loci were utilized in this research, and both grafted and non-grafted individuals were included in this study. Five closely related clones were identified, which represent a singular, polyclonal marron variety, found in all three cultivation areas. Furthermore, many hybrids, a result of breeding between cultivated and wild chestnuts, have been found. Analyzed semi-wild orchards defined by a diverse genetic structure, represent a hotspot for further selection and could result in creation of locally adapted, high-yielding varieties.

## Introduction

While the cultivated plants are characterized by limited genetic diversity, resulting from the process of domestication^1^, their wild relatives display greater genetic variability, which can be utilized as a source of genes lost during this process^2^. On the other hand, genes originating from cultivated populations have been shown to influence the genetic structure of wild populations in numerous species^3^. This gene flow between wild populations and cultivated varieties of the same or closely related species is well-known^4^ and has been observed when cultivated varieties are planted within the natural range of their wild relatives^5–7^, with spontaneous hybrids known to form their own distinct populations and persist in the wild^8,9^. While this introgression can lead to the extinction of the wild lineages^10^, alternatively it may cause increase of genetic variation and the creation of novel adaptations^11^.

A species illustrating the complexity of the cultivated-to-wild gene flow is the sweet chestnut (*Castanea sativa* Mill.), which has spread both naturally and aided by humans throughout the Mediterranean region and Central Europe^12^, especially during the time of the Roman Empire^13^. Together with olive tree^14,15^ and grapevine^16,17^, this species played a crucial role in sustaining the Mediterranean and south-Alpine communities, particularly during the Middle Ages^13,18^. As a result, countries with natural populations of this tree have created and cultivated their own local varieties^19,20^. Although a multi-purpose species used for its fruits, wood, honey and tannins^21^, selection of the cultivated varieties of sweet chestnut primarily focused on traits pertaining to fruit^22^. As a result, varieties known as marrons, characterized by sweet fruit of high quality, were developed and are still subjected to the official standards in both France and Italy^19,23^.

Due to the importance of chestnuts, the diversity of the wild chestnut germplasm in Europe has been extensively researched using the genomic (gSSRs)^24,25^ and genic EST-derived (EST-SSRs)^26,27^ markers, as well as chloroplast DNA (cpDNA)^28^. Three main gene pools had been revealed, coinciding with major countries of production^28,29^ and genetic divergence between the eastern (Greek and Turkish) and western (Italian and Spanish) populations was determined, coinciding with the glacial refugia of the south European flora^30^. In addition, a trend of genetic separation of populations from north to south was noted^26^, but general haplotypic diversity is low, most likely due to a strong human impact on the species^25,28^. Human influence on the species is also evident in the high number of marron varieties, with over 300 known in Italy alone^31^. The diversity of European marron varieties has been analyzed using gSSRs in Switzerland^32^, Italy^12,33–35^ and the Iberian Peninsula^36–41^, as well as using the EST-SSRs markers in Italy and Spain^42^. In addition, researchers looked into introgression and hybridization between cultivated and wild populations^3,22,35,43–45^.

One of the chestnut growing regions of Europe is the north-eastern coast of the Adriatic, with the majority of orchards in the regions of Istria and Kvarner, characterized by Sub-Mediterranean climate, which provides optimal conditions for both, cultivated orchards, as well as native stands of wild chestnuts. As a result, production thrives and marron exports from these regions have been recorded as early as the seventeenth century^46^. In addition, both wild and cultivated chestnuts have become an integral part of the local folklore and customs and festivals, such as “Lovranska marunada” (http://www.marunada-lovran.com) which take place every Autumn. Since family-owned orchards are located within the natural populations, both wild and cultivated trees can freely intermix. In addition, locally known “marušnjak” trees, hybrids between the two, are being cultivated in the same manner as the grafted clones, albeit on a smaller scale^45^. Nowadays, the production of marrons is being popularized and neglected or abandoned orchards are being revitalized^19^.

This newly rekindled interest for marrons has brought back the traditional varieties into the focus of the producers. In Istria and Kvarner regions, three toponymic marron varieties are known locally: the ‘Lovran Marron’, the ‘Lovrin Marron’ and the ‘Cres Marron’. While ‘Lovran Marron’ is very well known and commonly grafted within the orchards, the remaining two varieties are largely overlooked, and their cultivation is on a much smaller scale. Although known by the local producers, data on the variability and characteristics of the marrons in this region is limited, with previous research only conducted in the Lovran area^45,47,48^. As a result, the dominance of one genotype was determined, but studies were conducted on a small number of individuals using a limited set of markers and thus revealed only a part of the genetic diversity. In addition, further research into morphological and chemical variability of marrons in the Lovran area was conducted, uncovering that the dominant genotype can be considered a marron variety, according to both French and Italian standards^19,20^. Unlike the orchards of Lovran, orchards in Lovrin and Cres have never been researched, thus opening the possibility for additional varieties to be confirmed. In addition, orchards in all three cultivation areas are located within or in the vicinity of wild chestnut populations, enabling gene flow between the wild and the cultivated trees. This introgression of genes from cultivated trees could alter the genetic structure of the wild populations and produce hybrids, which could spread through both wild and cultivated populations.

Due to the unique combination of actively cultivated and neglected orchards as well as wild populations in a relatively small area, chestnut varieties from the northern Adriatic provide a unique opportunity to study the process of gene flow between populations of a partially cultivated tree species. Therefore, the aim of this research was to determine and describe three cultivation regions of sweet chestnuts in the regions of Lovran, Lovrin and island Cres as possible selection zones for traditional varieties in the past, with the possibility of determining and describing three varieties locally believed to exist. Six genomic simple sequence repeat (gSSR) and nine EST-derived SSR (EST-SSR) markers were used to explore the population structure of the orchards, with the aim of confirming the existence of three distinct varieties within the 219 sampled trees which included potential marron clones within 95 visibly grafted trees. Additionally, we analyzed potential hybrids between cultivated and wild trees, as well as ungrafted trees within orchards, which could have spontaneously spread into them from surrounding forests.

## Results

### Microsatellite diversity

From the total of 219 individuals included in the research (ALL), 141 were classified as non-redundant multi-locus genotypes (MLG). While the ALL group encompassed all sampled trees, the MLG group consisted of only those genotypes which were distinct. Microsatellite diversity was therefore analyzed on both levels, for all individuals (ALL) and non-redundant genotypes (MLG).

All 15 gSSR and EST-SSR markers were polymorphic, with a total of 86 alleles revealed, 52 for gSSR and 34 for EST-SSR respectively (Table 1). The number of alleles (*N*_*a*_) varied between gSSR and EST-SSR markers, from 12 (CsCAT6) to two (FIRO30). gSSR markers have shown to be significantly more variable, with average alleles number of 8.667, in comparison to 3.778 for EST-SSR markers (Table 1). The effective number of alleles (*N*_*e*_) was also higher for gSSRs in both ALL and MLG, with mean values of 2.444 and 2.695, respectively, but has not shown significant differences between the two groups of markers. Number of minor alleles (*N*_*ma*_), with frequency below 5%, was significantly higher for the gSSRs, with average value of 5.500 for ALL and 5.167 for MLG, in comparison to the EST-SSR values of 0.778 and 0.667, respectively.

**Table 1:** Allelic diversity of six SSR loci and nine EST-SSRs for all individuals (ALL) and the non-redundant genotypes’ group (MLG).

No.	Locus	Type	Motif	Reference	Size range	All				MLG		
						*N*_*a*_	*N*_*ma*_	*N*_*e*_	*PIC*	*N*_*ma*_	*N*_*e*_	*PIC*
1	CsCAT6	SSR	TC (cca 143 bp)	Marinoni et al.^52^	158–194	12	9	2.963	0.602	9	3.392	0.639
2	CsCAT1	SSR	(TG)5TA(TG)24 (cca 220 bp)	Marinoni et al.^52^	194–228	9	5	3.662	0.678	5	4.073	0.682
3	CsCAT16	SSR	TC (cca 143 bp)	Marinoni et al.^52^	121–217	10	6	3.564	0.668	5	4.167	0.691
4	CsCAT3	SSR	AG (cca 224 bp)	Marinoni et al.^52^	212–256	11	7	3.934	0.708	6	5.373	0.785
5	EMCs15	SSR	CAC (cca 90 bp)	Buck et al.^87^	81–93	5	3	1.242	0.187	3	1.396	0.013
6	OAL	SSR	(CT)16AGT(CT)2 (cca 300 bp)	Gobbin et al.^32^	299–331	5	3	1.984	0.406	3	1.840	0.412
7	WAG11	EST-SSR	CT (235–252 bp)	Durand et al.^88^	217–231	3	1	1.361	0.233	1	1.593	0.326
8	PIE233	EST-SSR	CCA (162–168 bp)	Durand et al.^88^	162–168	3	0	2.322	0.488	0	2.352	0.504
9	PIE228	EST-SSR	AGA (177–196 bp)	Durand et al.^88^	175–193	6	1	3.720	0.684	1	4.515	0.718
10	PIE227	EST-SSR	TGG (154–179 bp)	Durand et al.^88^	158–179	5	0	2.979	0.603	0	3.671	0.664
11	WAG004	EST-SSR	TTC (260–271 bp)	Durand et al.^88^	260–272	4	2	1.874	0.392	1	1.610	0.368
12	PORO009	EST-SSR	AG (122–140 bp)	Durand et al.^88^	120–128	4	0	3.251	0.634	0	3.679	0.671
13	FIRO30	EST-SSR	AG (168–173 bp)	Durand et al.^88^	168–172	2	1	1.056	0.052	1	1.089	0.052
14	PORO26	EST-SSR	TC (137–148 bp)	Durand et al.^88^	139–151	4	2	1.902	0.376	2	1.736	0.337
15	PIE260	EST-SSR	AG (154–168 bp)	Durand et al.^88^	153–165	3	0	2.088	0.435	0	1.990	0.439
gSSR						8.667	5.500	2.444	0.542	5.167	2.695	0.537
EST-SSR						3.778	0.778	1.972	0.433	0.667	2.029	0.453
*P*_Wilcoxon_						**	**	ns	ns	**	ns	ns

*N*_*a*_ number of alleles per locus, *N*_*ma*_ number of minor alleles, *N*_*e*_ effective number of alleles, *PIC* Polymorphism Information Content.

*PIC* values calculated for ALL ranged from 0.052 (FIRO30) to 0.708 (CsCAT3), whereas the MLG demonstrated mostly higher *PIC* values, with range between 0.013 (EMCs15) and 0.785 (CsCAT3), with three markers showing lower *PIC* values for MLG than ALL (WAG004, PORO26, EMCs15) and marker FIRO30 having the same *PIC* value in both ALL and MLG (0.052). Most informative loci for the MLG were PIE228 and CsCAT3, with *PIC* values above 0.7, whereas eight markers (OAL NED, PIE260, PIE233, CsCAT6, PIE227, PORO009, CsCAT1 and CsCAT16) were moderately informative, with *PIC* values within the range 0.412–0.691. When observing all individuals (ALL), only CsCAT3 marker was highly informative (0.708), whereas moderately informative loci remained the same as in MLG. No statistically significant differences in *PIC* values between the EST-SSR and gSSR marker groups were determined.

### Population diversity and clonality

**Figure 1:**
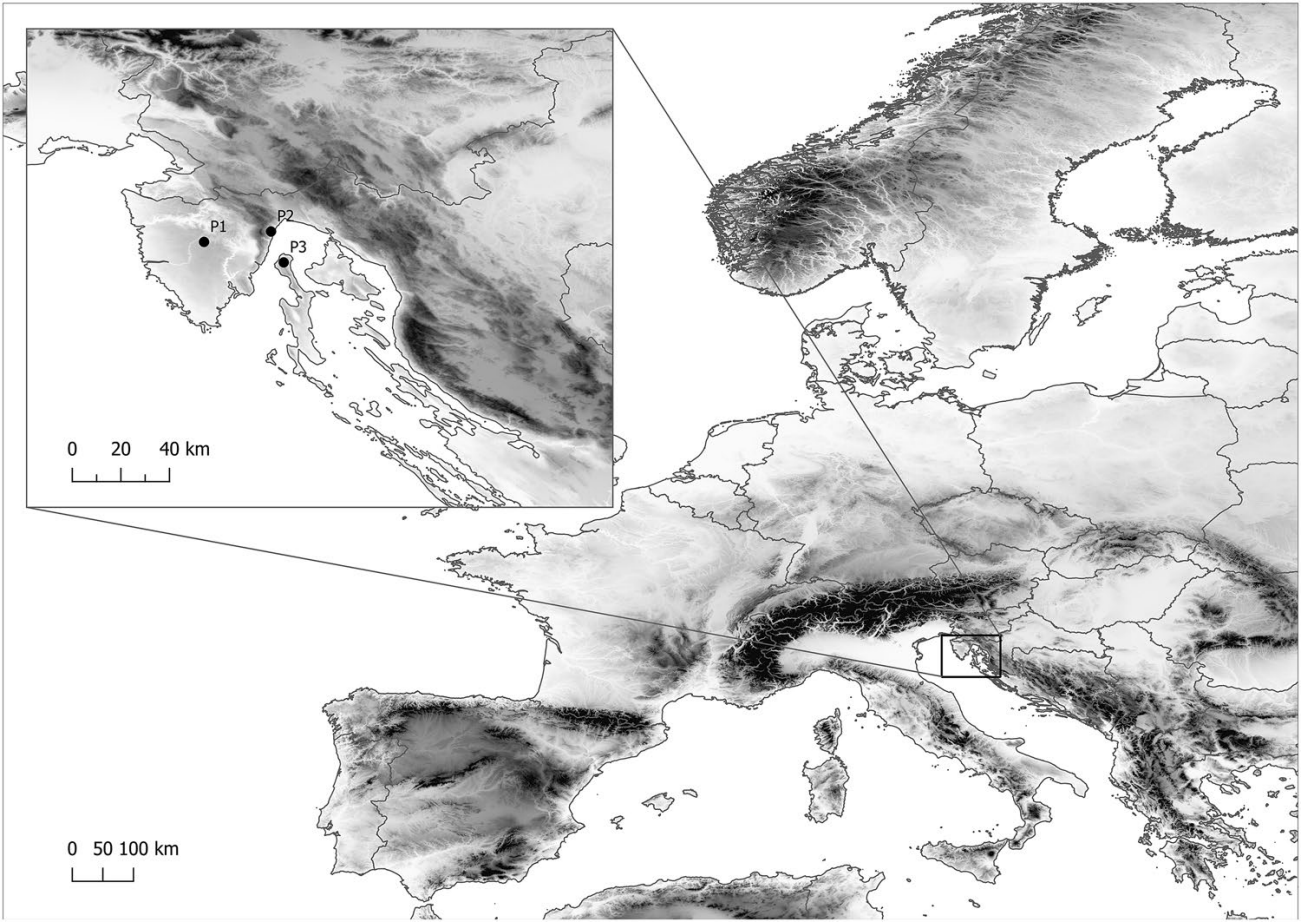
Geographical distribution of the three marron producing areas in the northern Adriatic region. Each of the locations with the orchards was considered a singular population, with ‘Lovrin Marron’ orchards named “Population 1” (P1), ‘Lovran Marron’ orchards as “Population 2” (P2) and ‘Cres Marron’ as “Population 3” (P3). In total, 219 trees were sampled, 44 trees in P1, 83 in P2 and 92 in P3. The map was generated using QGIS 3.10.7 (https://qgis.org/).

To observe the allelic diversity on the population level, three groups of individuals were considered: P1 (Lovrin), P2 (Lovran) and P3 (Cres), with 44, 83 and 92 individuals sampled in each group, respectively (Table 2). Within these populations (Fig. 1), grafted individuals were easily spotted and noted as likely of clonal origin. Within each population several non-grafted or individuals of undetermined origin were found as well. Therefore, we utilized the allelic analysis to test for levels of clonality within each population. The highest number of alleles per locus (*N*_*av*_) was determined for P1 (5.267), as well as the highest levels of allelic richness (*N*_*ar*_) (5.267). In addition, P1 was characterized by the largest number of private alleles (*N*_*pra*_) (15), compared to significantly lower numbers for P2 (three) and P3 (four).

**Table 2:** Parameters of allelic and clonal diversity of the three sampled populations: P1—Lovrin; P2—Lovran; P3—Cres.

Pop	Locality	*n*	*N*_*av*_	*N*_*ar*_	*N*_*pra*_	*N*_*g*_	*n*_*c*(*M001*)_	*n*_*c*(*M001*)_ (%)	*n*_*c*(*M002–M005*)_	*n*_*c*(*M002–M005*)_ (%)	*N*_*ug*_	*n*_*c*(*ug*)_ (%)	*R*	D*	*H*_*O*_	*H*_*E*_
P1	Lovrin	44	5.267	5.267	15	44	0	0.00	1	2.27	43	97.73	1.000	1.000	0.609	0.571
P2	Lovran	83	3.467	2.942	3	17	65	78.31	5	6.02	13	15.66	0.195	0.388	0.737	0.490
P3	Cres	92	3.933	3.665	4	84	3	3.26	9	9.78	80	86.96	0.912	0.996	0.580	0.516
Total		219			22	145	68		15		136					

*n* total sample size, *N*_*av*_ average number of alleles per locus, *N*_*ar*_ allelic richness, *N*_*pra*_ number of private alleles, *N*_*g*_ number of distinct genotypes, *n*_*c(M001)*_ clonal size of the clone M001, *n*_*c(M001)*_* (%)* % of the samples belonging to the M001, *n*_*c(M002–M005)*_ clonal size of the clones M002–M005, *n*_*c(M002–M005)*_* (%)* % of the samples belonging to the M002–M005, *N*_*ug*_ number of unique genotypes, *n*_*c(ug)*_* (%)* % of the samples belonging to unique genotypes, *R* genotypic richness, D* Simpson's complement index, *H*_*O*_ observed heterozygosity, *H*_*E*_ expected heterozygosity.

A total of 141 distinct multi-locus genotypes (*N*_*g*_) were identified, with the highest number of distinct genotypes found in P3 (84), followed by P1 (44) and P2 (17). The most common genotypes, with all individuals showing visible graft joints, were those belonging to the M001–M005 group, with the prominent predominance of the M001 clone. This genotype was found only in P2 and P3 and accounted for 78.31% and 3.26% of the total sample size in these populations, respectively. Clones M002–M005 accounted for 2.27%, 6.02% and 9.78% of the total sample size in P1, P2 and P3, respectively. Due to the predominance and clear clonal origin, the clonal group M001–M005 was named the “Marron clones” and analyzed in depth. Number of unique genotypes (*N*_*ug*_) was 136, with the majority found in P3 (80), accounting for 86.96% of the total sample in that population. In addition, 43 unique genotypes in P1 represented 97.73% of the total sample size, signifying these two populations as highly diverse. In that population only one out of the 44 individuals, was a ramet of a clone found in the M002–M005 clone group, i.e., the remaining 43 were individuals not found anywhere else. On the other hand, P2 was characterized by having only 13 unique genotypes, representing 15.66% of the total sample size. In addition, this population was noted to have the highest number of grafted individuals. This is in agreement with the very high number of P2 individuals belonging to a single clone, M001, and further supported by values of genotypic richness (*R*) and Simpson’s complement index (D*), both of which demonstrated low values in P2, 0.195 and 0.388, respectively. In comparison, the most diverse population, P1, scored values of 1.000 for both parameters. In all three populations, expected heterozygosity (*H*_*E*_) was lower than the observed (*H*_*O*_), indicating high levels of genetic variability, in accordance with previously mentioned results.

### Genetic distance; population differentiation and structure

Genetic differentiation values, *F*_*ST*,_ were statistically significant between all population pairs. The highest *F*_*ST*_ value was noted between the P1 and P2 populations (*F*_*ST*_ = 0.151; p < 0.001), and a similar *F*_*ST*_ value was noted between P2 and P3 (*F*_*ST*_ = 0.111; p < 0.001). The lowest value of *F*_*ST*_ was between P1 and P3 (*F*_ST_ = 0.066; p < 0.001). In addition, analysis of molecular variance (AMOVA) showed statistically significant differences among populations, with 10.83% of total variation attributed to inter-population variability and 89.17% of variability resulting from intra-population variability (Supplementary Table S1).

**Figure 2:**
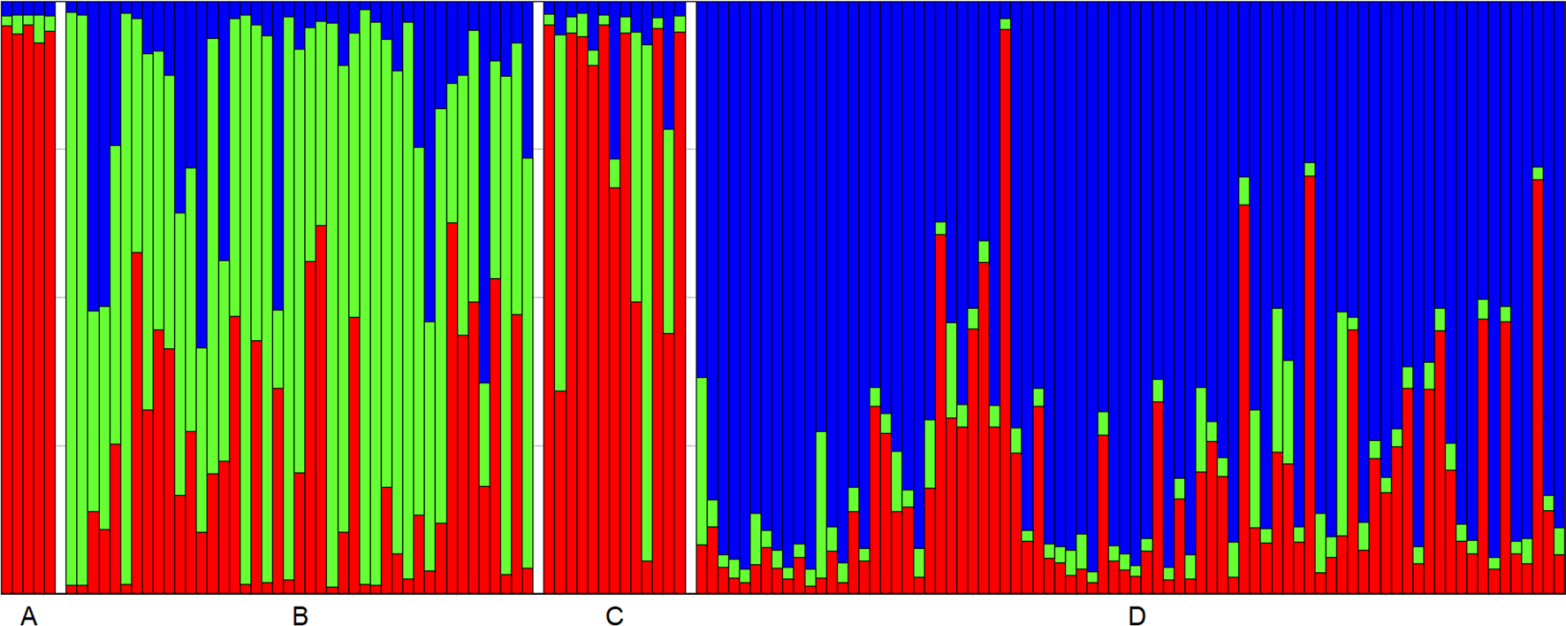
Genetic structure of 141 sweet chestnut multi-locus genotypes (MLG) as estimated by STRUCTURE at K = 3. Each MLG is represented by a vertical line and each cluster by a different color: Cluster I in red, Cluster II in green, Cluster III in blue. Letters **A**, **B**, **C** and **D** are as follows: Group A represents marron genotypes M001-M005, Group B non-redundant genotypes in P1, Group C non-redundant genotypes in P2 and Group D non-redundant genotypes in P3.

The STRUCTURE was used to study the genotype distribution and genetic relation among the 141 distinct multi-locus genotypes analyzed in this research. The optimal number of genetic clusters (K) was K = 3, determined both by calculating ΔK^49^ (Supplementary Fig. S1) and MedMeaK, MaxMeaK, MedMedK and MaxMedK^50^ (Supplementary Fig. S2). Genotypes M001–M005 with the majority of individuals from population 2 were assigned to Cluster I (Fig. 2), with a total of 14 genotypes in this cluster. Cluster II encompassed 15 individuals and was predominantly formed of individuals from population P1, whereas the majority of individuals from population P3 were assigned to Cluster III, with a total of 46 individuals forming this cluster. Population P1 was the most admixed, with 29 (67.44%) of the individuals having the membership probability Q < 75% for each cluster, whereas population P2 was the least admixed with only four individuals (30.77%) of mixed origin. Overall highest number of mixed-origin individuals—28, displayed the membership probability Q < 75% for Cluster III and belonged to the population P3.

**Figure 3:**
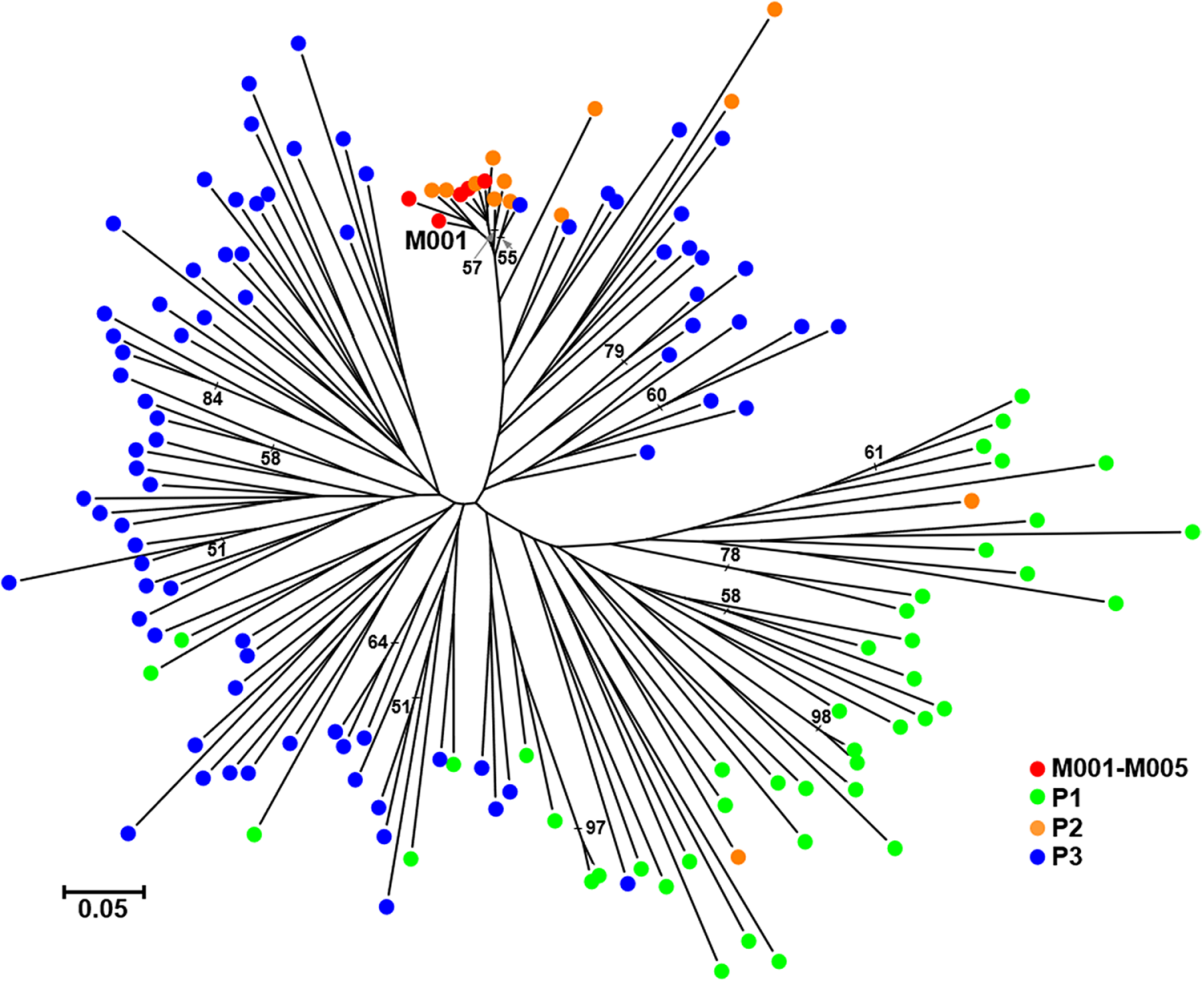
Neighbor-joining tree based on microsatellite marker data of 141 sweet chestnut multi-locus genotypes. The clonal group (M001–M005) or the population of origin (P1, P2, P3) of each MLG are indicated on branches of the tree. The numbers above branches indicate bootstrap support values over 50% in 1000 pseudoreplicates. The tree was visualized using MEGA7 (https://www.megasoftware.net/).

To further investigate the population structure of the researched genotypes, an unrooted neighbour-joining tree was created (Fig. 3). The results support results from the previously described analysis, with genotype group M001–M005 clustered together with individuals belonging to P2. Populations P1 and P3 separated into two distinct clusters with only a small overlap between them.

### Relatedness

**Figure 4:**
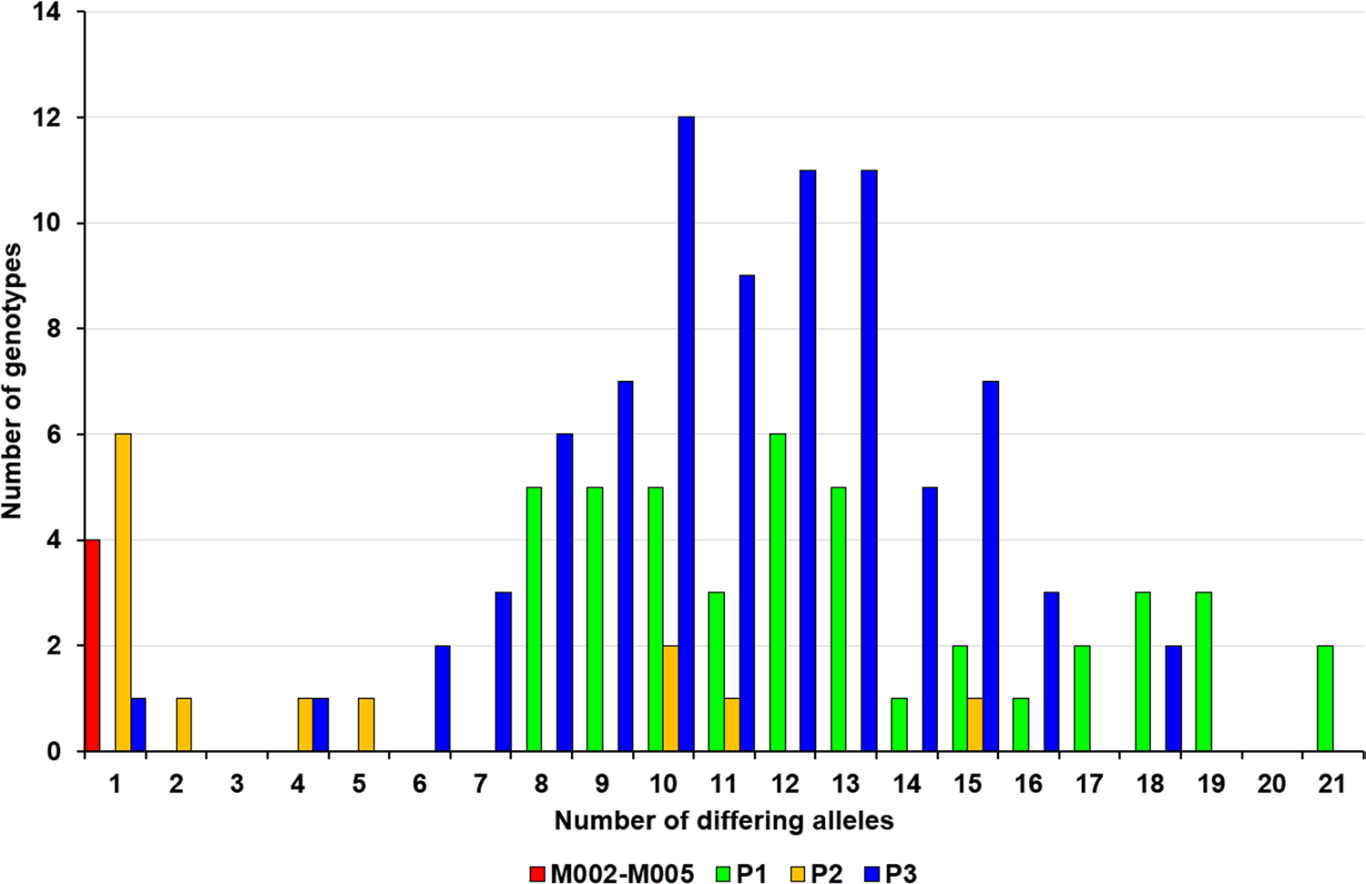
Histogram of number of different alleles based on 15 polymorphic microsatellites between the M001 genotype and all other analyzed genotypes.

Relatedness between the most common genotype M001 and the remaining 140 unique multi-locus genotypes was analyzed and the number of differentiating alleles was determined (Fig. 4). P2 demonstrated the lowest number of differentiating alleles, in addition to the lowest number of unique genotypes. In this population, six MLGs were differentiated by a single allele, with five other genotypes differentiated by two, four, five, 11 and 15 alleles respectively. Additionally, two MLGs were differentiated by 10 alleles. P3, on the other hand, was the most diverse, with 60 out of 80 unique MLGs differentiated by more than 10 alleles and only two MLGs having less than five different alleles. MLGs from P1 differentiated with at least eight alleles, with the differentiating alleles’ number ranging from eight to 21. Additionally, marron group M002–M005, with four analyzed individuals, demonstrated a single differentiating allele, proving close genetic similarity to M001.

**Figure 5:**
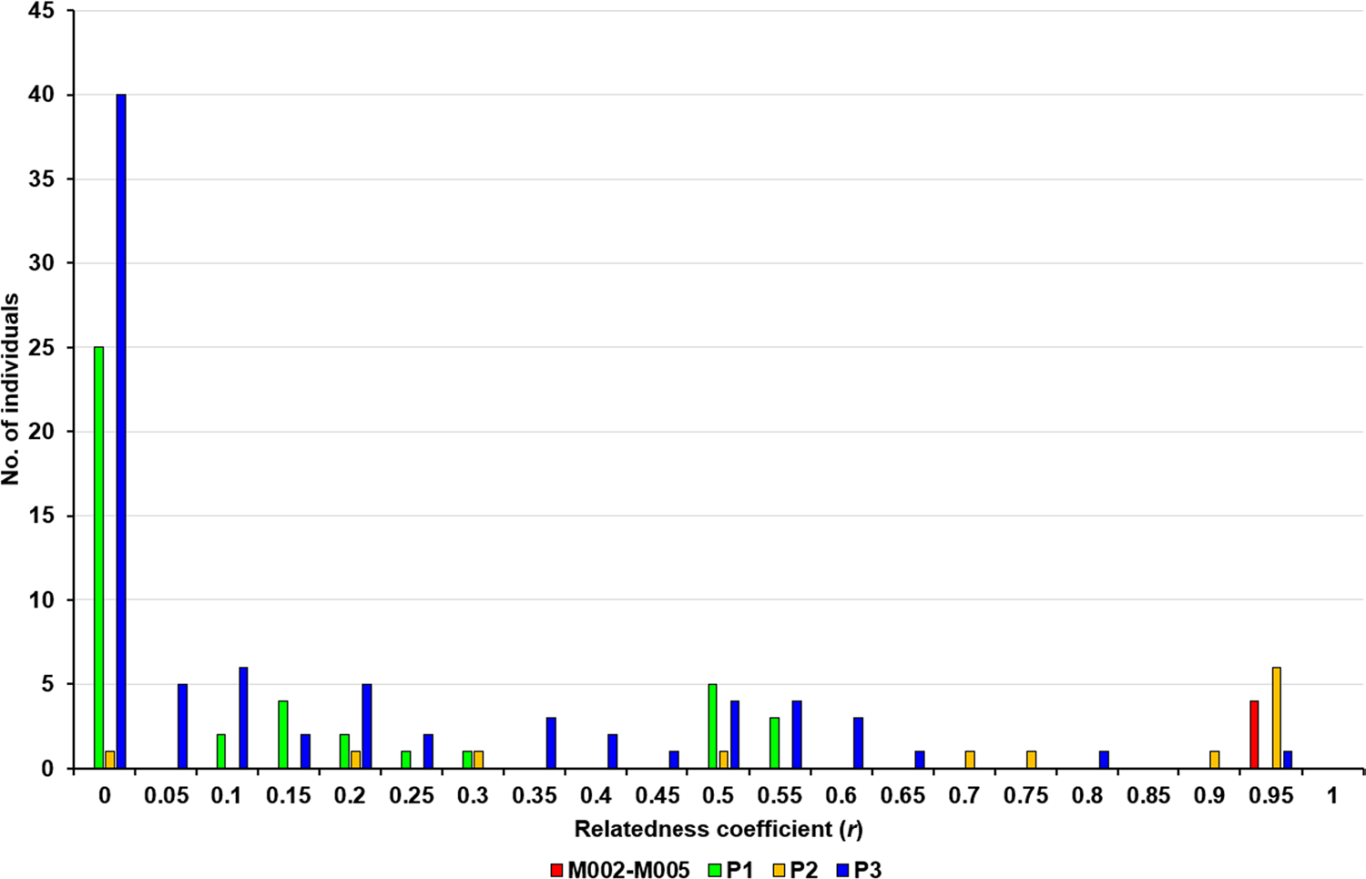
Histogram of coefficients of relatedness (r) based on 15 polymorphic microsatellites between the M001 genotype and all other analyzed genotypes.

The coefficient of relatedness (*r*) was calculated for all genotypes relative to genotype M001 (Fig. 5) and ranged from 0.00 to 0.95. As expected, the highest *r* value − 0.95, was obtained for the M002–M005 group. In addition, the same *r* was found for six individuals from P2 and one from P3. All 43 genotypes from P1 had *r* in the range between 0.00 and 0.55, indicating the lowest relatedness to M001. On the other hand, nine genotypes from P2 had *r* greater than or equal to 0.70, indicating high relatedness to M001. The low relatedness was also observed in P3, where only two individuals had an *r* greater than or equal to 0.70. A total of 66 individuals were genetically unrelated to M001 (*r* = 0.00), with 25 (58.14%) found in P1, one (7.69%) in P2, and 40 in P3 (50.00%).

**Figure 6:**
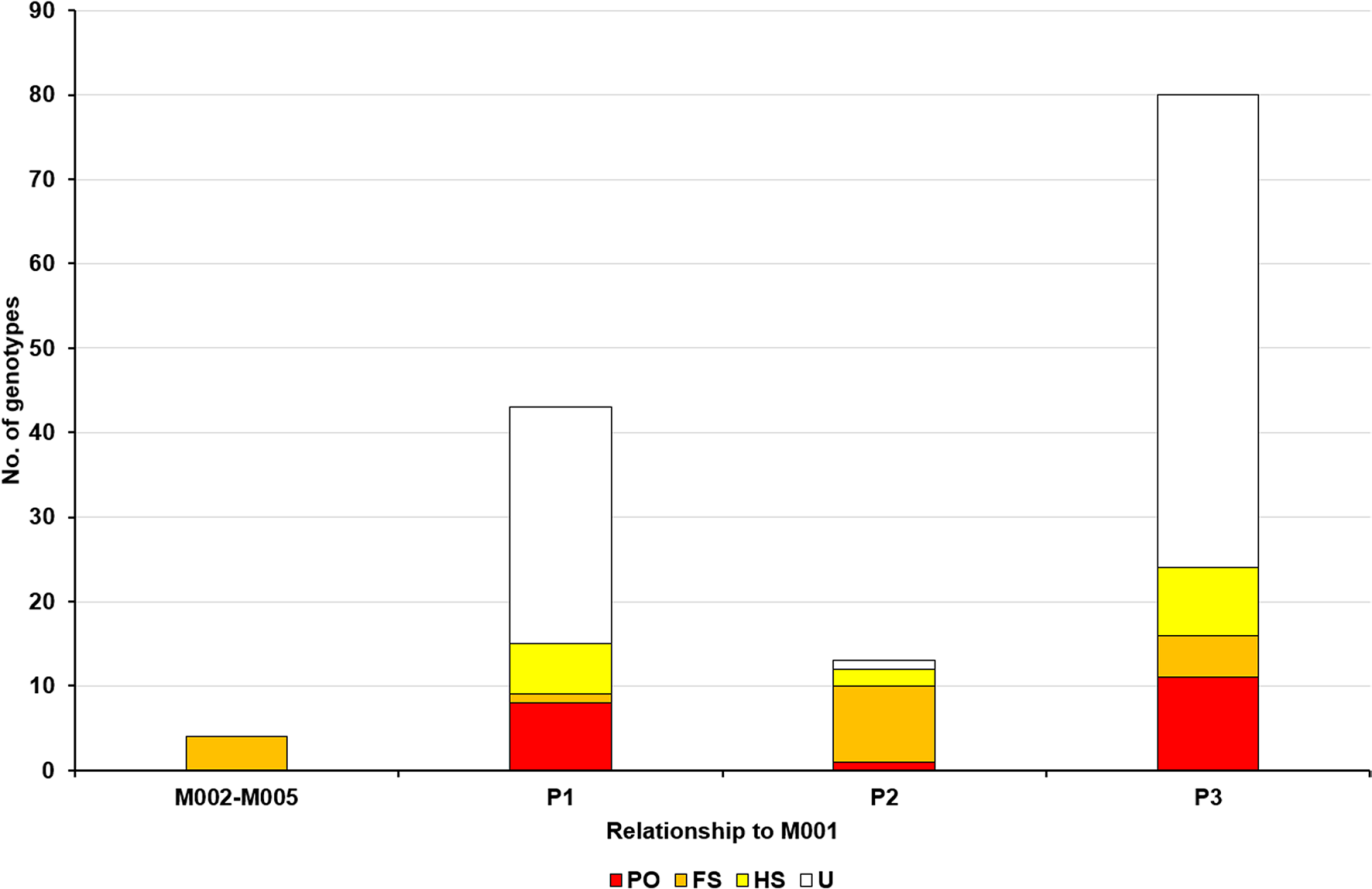
Histogram of kinship categories between the analyzed genets of sweet chestnut and the most common genotype M001. Four pedigree relationships are represented by colors in the columns: unrelated (U) in white, half-siblings (HS) in yellow, full-siblings (FS) in orange and parent-offspring (PO) in red.

Furthermore, we identified a putative pedigree relationship between M001 and the rest of the genotypes based on the highest likelihood among four possible relationships: unrelated (U), half-siblings (HS), full-siblings (FS), and parent-offspring (PO) using ML-Relate (Fig. 6). All four genotypes of the M002–M005 group were classified as full-siblings (FS). P2 showed similar results, with 69.00% of genotypes classified as FS, two as half-siblings (HS) and only one genotype as parent/offspring (PO). P1 and P3 were far less related to M001, with 28 (65.12%) and 56 (70.00%) of the genotypes classified as unrelated (U), respectively. One individual from P1 and five from P3 were classified as FS with M001, eight from P1 (18.6%) and 11 from P3 (13.75%) as PO, and six from P1 (13.95%) and eight from P3 (10%) as HS.

## Discussion

Our results confirmed the high effectiveness of both gSSR and EST-SSR markers in describing the genetic diversity of sweet chestnut, as was previously reported for wild populations^24–26,45,51^ and cultivated varieties^12,22,33,34,36,40,42^. The most and least informative gSSR loci were CsCAT3 (*PIC* = 0.785) and EMCs15 (*PIC* = 0.013), respectively, which was expected as the same was reported previously^22,24,36,37^. The *N*_*a*_ values obtained in this research (8.67 for gSSR and 3.78 for EST-SSR) fall within the range of those previously reported in southern Europe for both gSSR and EST-SSR markers^12,22,26,34,36,40,42,52^, with ranges of 4.75–13.25 and 0.78–4.00, respectively. The data differences between gSSR and EST-SSR marker groups stem from the different regions of genomes they assess. While SSRs are located in introns or noncoding regions of the DNA, EST-SSRs are particularly associated with functional genes^53^. EST-SSRs are more conserved due to their linkage to a certain gene and are thus a cost-effective and labour-saving option in assessing the genetic diversity within or adjacent to a certain gene, whereas gSSRs' abundance in the genome, easy reproducibility, and high polymorphism makes them specifically suitable for germplasm characterization, variety identification and paternity analysis^53–56^.

Although considered to be orchards at the beginning of our research, P1 and P3 demonstrated genetic diversity levels more akin to those of wild chestnut populations, with expected and observed levels of heterozygosity and genotypic richness values similar to the previously reported in Spain^26,30,51^ and Switzerland^25^, and slightly lower than those reported in Bulgaria^57^, Italy^30^ and Greece^26^. In comparison to populations from Asia Minor and the Black Sea region, defined by the highest genetic variability of chestnut populations resulting from being at the meeting point of two glacial refugia^30,58^, values obtained in our research were understandably lower. As a result of land abandonment and depopulation in the twentieth century^12,19^, these orchards have been left unattended and are, to a large extent, overgrown by natural vegetation. The influx of wild chestnut genes has increased the diversity of these populations and nowadays populations P1 and P3 are genetically closer to wild chestnut stands than to cultivated orchards. Although clonal individuals were found in P3, the majority of noted grafted trees were dead and, together with the lack of newly grafted individuals, indicated these clones are likely the last individuals from the once cultivated orchard. Land abandonment to this degree has been shown to influence spontaneous landscape restoration and rewilding, particularly in the Mediterranean basin^59^ and, in synergy with the differences in the habitat conditions, certainly affected the levels of genetic diversity noted in P1 and P3.

Unlike the naturalized state of populations P1 and P3, population P2 demonstrated low genetic diversity akin to an orchard. Pronounced human influence and longer periods of vegetative propagation present in P2 likely maintained the low genetic diversity of this population, as previously reported by Mattioni et al*.*^29^. Most of these orchards have been cultivated by local families in the Lovran area, with the knowledge of grafting techniques of the ‘Lovran Marron’ passed on for generations^60^. This is evident in the high number of clonal genotypes, with 70 individuals belonging to the M001 genotype and M002–M005 genotype group. Low allelic diversity of the population P2 is further illustrated by only 13 distinct genotypes found, as well as very low values of genotypic richness (0.195) and Simpson's complement index (0.388). This suggests the Lovran area is the original cultivation zone of marrons in the North Adriatic region, from which the culture of chestnut spread to Lovrin and Cres, albeit to a lesser degree.

The results of AMOVA indicate 89.17% of total variance to be assigned to variation within each population, whereas only 10.83% referred to the variation among populations in accordance with other research on sweet chestnut populations in Europe^29,30,57^. These values indicate a lack of intensive cultivation and high genetic diversity on population level, although great differences in genetic diversity and clonality between the populations exist, with population P2 clearly demonstrating the strongest influence of clonality on its genetic diversity. In addition, these high levels of clonality in P2 have led to significant differences to be noticeable between P2 and the remaining two populations. However, as P1 and P3 are characterized by a larger number of distinct genotypes, and similarly low degrees of human interference, these two populations are genetically statistically significantly different. Variable conditions in the environment could have influenced these differences since heterogeneity of habitat is known to play a major role in genetic differentiation^61,62^. Almost a complete lack of human influence on the P1 and limited human influence in P3 meant that the natural processes of selection, with alleles providing adaptation to specifics of the habitats of each population^3,63^ were far more important than the selection brought on by cultivation. Alternatively, P1 and P3 could be the result of two separately introduced gene pools, as opposed to a series of natural colonization events^45^. As pollen analysis in the eastern Adriatic revealed, anthropogenic influence on native species was significant, dating back to the times of Greek and Roman colonization^64,65^.

The most common genotype overall was the genotype M001, found in populations P2 and P3, with the second most common genotype being M002, with all five individuals found in P3. Ten individuals were assigned to genotypes M003–M005 and were distributed in all three populations. Genotype M001 was predominantly found in the cultivation area of P2, which was previously investigated by Poljak et al.^45^. In this previous research, ten markers were used to explore the structure of local orchards and the predominance of one genotype was determined. Out of those ten markers, six were also used in this research and have revealed the most common genotype observed in both the research by Poljak et al.^45^ and this research to be the same. Male-sterile and unable to pollinate, M001 nonetheless had proven to differ in a single allele from the M002–M005 clones, demonstrating a full-sibling relationship to them. Such relationship can be the result of one of two possible events in cultivation history of the clones. The likelier theory is that of the shared ancestor, i.e., the existence of ancestral polyclonality. This theory is based on the highly probable cross-pollinations within the ancient marrons, present in the Lovran area, before the male-sterility arose. This would have led to small genetic differences, and ultimately to inbreeding, evident as the present-day-male-sterility, as previously proven for other plant species^66,67^, as well as animals^68^. The selection that must have occurred favored individuals with appreciable fruit traits and this, in addition to male-sterility, remains even today an important trait in discerning the traditional variety known as ‘Lovran Marron’. Considering all five genotypes have developed a common phenotype and are, due to common methods of vegetative propagation, cultivated in the same manner, they have continued to co-exist and can be considered a singular, polyclonal variety ‘Lovran Marron’. Similar results were reported for traditional Portuguese olive (*Olea europaea* L.) variety ‘Galega Vulgar’^69^ and Croatian varieties ‘Lastovka’, ‘Oblica’ and ‘Drobnica’^70^, with common polyclonal ancestry also revealing Croatian variety ‘Bjelica’ and Montenegrin variety ‘Žutica’ to be synonymous^71^. Based on these facts, ‘Lovrin Marron’ and ‘Cres Marron’ should not be considered different varieties; rather, all genotypes from M001–M005 should be considered as a singular, polyclonal variety ‘Lovran Marron’. Although not identical, these genotypes are cultivated in the same manner, further supporting the polyclonal theory. The alternative explanation of the relationship between the M001–M005 clones is the accumulation of somatic mutations, through which from one ancestral genotype (M001), other four MLGs would have developed. Such mutations can accumulate without affecting the phenotype since gSSR regions are neutrally evolving and intrinsically very variable^72^. However, this theory is less likely, due to the relatively short time the mutations could have accumulated, of only three centuries, as opposed to ancient varieties of olives, in which such occurrences are well-documented^73^. On the other hand, sexual propagation and subsequent grafting has been shown to influence intravarietal variability of Iberian chestnut varieties^74^. Here, chestnut growers planted seeds of known varieties and, when seedlings were found to produce nuts with identical parameters, cultivated them under the same name, increasing intravarietal variability along the way. Alternatively, seedlings that produced seeds with different traits were named as a new variety and further cultivated by clonal propagation. This was evidenced by a frequent parent–offspring relationship between the main variety and its generative offspring. However, in this study, this theory is less likely because clones M002–M005 differ from the most common clone (M001) in a single allele, which excludes the possibility that they are its offspring resulting from a cross with wild chestnut.

Alongside with grafted, now proven clonal individuals in P2, non-grafted individuals related to M001 were found within the P2. These individuals are locally known as “marušnjak” trees and represent the long history of breeding between the cultivated and wild individuals, i.e., M001 progeny pollinated by a wild parent. These individuals are found in both wild populations^45^ and within orchards and their presence created the opportunity for a second wave of human selection, particularly considering the intermediate values of both leaf and fruit morphometric traits and chemical composition of these “marušnjak” fruits^19,20^. In turn, they could prove to be a valuable source of genetic variability, resulting from possible hybrid vigor^75^. Unlike the population P2, populations P1 and P3 demonstrated lower relatedness to M001, with over 50% of all individuals in both populations demonstrating *r* value of 0.00 and less than 20% of individuals defined by parent–offspring relationship. These results could be attributed to the conditions noted in the field. In these populations, the orchards are mostly neglected, with surrounding forest vegetation and wild chestnut trees taking over. In addition, large numbers of visibly grafted but dead trees have been observed in P3. Overall, populations P1 and P3 can be considered as an intermediate state between a cultivated orchard and a wild population. In other words, characteristics of the genetic diversity in these populations bear witness to past cultivation efforts, in form of the grafted trees, whilst a large number of non-grafted trees represent the succession of the wild forest back into the neglected orchards. The long history of broadening the natural distribution of sweet chestnut and the domestication within the range of local population had influenced the germplasm diversity of Switzerland^25^, whereas intermixing of the cultivated genes with the surrounding wild populations influenced the populations of Spain^51^ and Italy^29^. In addition, in France^41^, Italy and Spain^38^, the interaction between the orchards and the wild populations can particularly be observed in the “instant domestication” cases, i.e., grafting of the spontaneously grown chestnuts, which diminishes the genetic differences between the orchard and the wild population.

Similar genetic interactions between cultivated and wild populations are not uncommon and have previously been noted for 12 of the 13 most important food crops in the world^76^, as well as genera *Beta* L.^77^, *Malus* Mill.^10^, *Medicago* L.^78^, *Cichorium* L.^8^, *Lactuca* L.^79^ and *Solanum* L.^80^. Although some authors express concerns about the negative impact of this gene flow on the extinction of the wild populations^4,81–83^, the occurrence of “pestification”^10,84,85^ and the spread of transgenes from the cultivated crops to wild populations^79^, hybridization may, in contrast, increase genetic variability of a population and promote the emergence of beneficial adaptations^11^. A similar occurrence has been previously reported for cultivated apple (*Malus domestica* Borkh.)^6^, as well as for cacao plant (*Theobroma cacao* L.)^86^.

Overall, the genetic structure of the three cultivation areas we analyzed in this research did not confirm the existence of three locally known varieties. Rather, one polyclonal variety was found and can be considered as ‘Lovran Marron’ variety. The origin of ‘Lovran Marron’ is most likely the result of cross-pollination between ancestral genotypes, which led to male-sterility noticeable today. The possibility of somatic mutations causing or, at least, influencing the genetic variability of the variety cannot be excluded but is less likely to have occurred in such a short span of time. Likewise, it is unlikely the M002–M005 clones are the offspring of the predominant M001 genotype and a wild pollinator since they differ from the most common clone in a single allele. In addition, the prevalence of grafted individuals in the Lovran area indicates this to be the original cultivation area from which chestnut culture most likely spread to Lovrin and Cres. The cultivation-associated genes also spread spontaneously into the wild populations surrounding the orchards, through seeds of ‘Lovran Marron’. This offspring continues to be cultivated, thus broadening the gene pool of the populations, albeit to various degrees. Through this, valuable alleles from wild populations might be reintroduced into the cultivated genotypes and boost their response to changes in the environment, as well as possibly create new and improved chestnut varieties.

## Materials and methods

### Study area and plant material

The research was conducted in the three known marron producing areas of the Sub-Mediterranean zone: Lovran and Lovrin in Istria, and on the island Cres, a part of the Kvarner region (Fig. 1). Three locally known toponymic marrons, one from each of the three regions, were included in this research. The best-known variety, the ‘Lovran Marron’, is cultivated in family-owned orchards on a large number of smaller, terraced plantations. The other two varieties, ‘Lovrin Marron’ and ‘Cres Marron’ are currently not cultivated on a larger scale and the orchards are located in the singular, flat areas, partially overgrown by the surrounding forest. Therefore, each of the locations with the orchards was considered a singular population, with ‘Lovrin Marron’ orchards named “Population 1” (P1), ‘Lovran Marron’ orchards as “Population 2” (P2) and ‘Cres Marron’ as “Population 3” (P3). In total, 219 trees were sampled, 44 trees in P1, 83 in P2 and 92 in P3. In each population, visibly grafted, still alive, trees were particularly noted in a field diary, with a total of one, 80 and 14 grafted trees found in P1, P2 and P3, respectively. All trees, visibly grafted or not, were marked with metal plates containing the number code of the individual tree.

For the DNA extraction and further analyses, fully developed buds were collected during the vegetation season of 2019 from well-lit branches in the crown of the trees. Buds were collected into filter bags and stored in plastic bags with silica gel to dry. Once in the laboratory, samples were kept inside the silica gel, at room temperature and in the dark until further analysis. The collection of plant material was carried out in accordance with relevant institutional, national, and international guidelines and legislation. Voucher specimens were identified by Igor Poljak and Antonio Vidaković and are deposited in the DEND Herbarium, Zagreb, Croatia (available at: http://dendherbarij.sumfak.unizg.hr/herbariumdend_en.html; Herbarium IDs: 05493-05509).

### DNA extraction and molecular characterization

Dried buds were used to extract total genomic DNA using the DNeasy Plant Mini Kit (Qiagen GmbH, Hilden, Germany) according to the manufacturer’s instructions with the addition of 1% PVP and 1% β-mercaptoethanol in lysis buffer. DNA concentration was determined with NanoDrop (Thermo Fisher Scientific, Waltham, Massachusetts, USA). For genetic analysis, six genomic microsatellite gSSR markers (CsCAT1, CsCAT3, CsCAT6, CsCAT16, OAL, EMCs15) developed for sweet chestnut^32,52,87^, and nine expressed sequence tag EST-SSR markers (WAG11, PIE233, PIE228, PIE227, WAG004, PORO009, FIRO030, PORO26, PIE260) produced for *Quercus petraea* (Matt.) Liebl. and *Q*. *robur* L.^88^ were used. Polymerase chain reactions (PCRs) were performed in a total volume of 20 μL containing 1 × PCR buffer, 1.5 mM MgCl_2_, 200 μM of each dNTPs, 5 μM forward and reverse primers, 0.5 U TaqHS polymerase (TaKaRa Bio Inc., Shiga, Japan) and 5 ng of template DNA. The resulting products were analyzed by capillary electrophoresis on an ABI 3730xL DNA analyzer (Applied Biosystems, Foster City, CA, USA) provided by Macrogen DNA service (Amsterdam, Netherlands), and alleles were scored using GeneMapper software version 4.0 (Applied Biosystems, Foster City, CA, USA).

### Data analysis

The number of distinct multi-locus genotypes (MLGs) in three sweet chestnut populations was identified using GenClone 2.0^89^.

For each microsatellite locus, the number of alleles per locus (*N*_*a*_), polymorphic information content (*PIC*), and probability of identity (*PI*) were calculated using Cervus v3.0^90^. The effective number of alleles (*N*_*e*_) was derived from the expected heterozygosity (*H*_*E*_) using the following formula: *N*_*e*_ = 1/(1 − *H*_*E*_). The alleles with a frequency lower than 0.05 (5%) were considered minor alleles. The parameters were calculated using all individual trees (*n* = 219) as well as non-redundant genotypes (i.e. unique MLGs; *n* = 141). To test the significance of the differences in *N*_*a*_, *N*_*ma*_ (number of minor alleles), *N*_*e*_ and *PIC* between EST-SSRs and gSSRs, a nonparametric Wilcoxon signed-rank test was performed using SAS v9.3^91^.

Allelic diversity of populations was assessed by calculating the average number of alleles per locus (*N*_*av*_), allelic richness (*N*_*ar*_), and the number of private alleles (*N*_*pra*_). We used FSTAT v2.9.3.2 software^92^ to calculate allelic richness (*N*_*ar*_; the number of alleles per locus regardless of sample size). Clonal diversity of populations was assessed by calculating the number of multi-locus genotypes (*N*_*c*_), genotypic richness (*R*)^93^ and Simpson's complement index (D*)^94,95^ using GenClone v2.0^89^. Genetic diversity of populations was assessed by estimating observed (*H*_*O*_) and expected (*H*_*E*_) heterozygosity using GENEPOP v4.0^96^, excluding redundant genotypes from each population.

Genetic distances between multi-locus genotypes were calculated in MICROSAT^97^ using proportion-of-shared-alleles distances (*D*_*psa*_)^98^. The Neighbor-joining tree was constructed using PHYLIP v3.698^99^. The tree was bootstrapped^100^ over 1000 replicates generated in MICROSAT and subsequently used in the NEIGHBOR and CONSENSE programs in PHYLIP.

Population differentiation was assessed by calculating pairwise *F*_*ST*_ estimates in FSTAT. *P* values were calculated after 10,000 random permutations. ARLEQUIN v3.5.2.2^101^ was used for the analysis of molecular variance (AMOVA)^102^ by partitioning total microsatellite diversity among and within sweet chestnut populations. The significance level of *φ*_*ST*_ was determined by a nonparametric randomization test with 10,000 permutations.

The genetic structure of sweet chestnut populations was assessed using STRUCTURE v2.3.4^103^. Thirty runs were performed with the number of clusters set from 1 to 11, on the Isabella computer cluster at the University of Zagreb (Croatia), University Computing Centre (SRCE). Each run consisted of a burn-in period of 200,000 steps followed by 10^6^ MCMC replicates using the admixture model with correlated allele frequencies. The optimal number of clusters was determined by calculating ΔK^49^ and MedMeaK, MaxMeaK, MedMedK and MaxMedK^50^ as implemented in StructureSelector^104^, which integrates the CLUMPAK program^105^ used to cluster and merge data from independent runs.

A maximum-likelihood method implemented in ML-Relate^106^ was used to calculate a pairwise estimate of the relatedness (*r*) of M001 to each tree across populations and to discriminate among four different pedigree relationships: unrelated (U), half-siblings (HS), full-siblings (FS), and parent-offspring (PO).

### Ethical approval

The collection of plant material was carried out in accordance with relevant institutional, national, and international guidelines and legislation.

## Supplementary Information

Supplementary Information.

## Data Availability

The allelic datasets analysed during the current study are available in the Zenodo repository, https://doi.org/10.5281/zenodo.6448096.
