# Supplementary material for: Gene flow between wild trees and cultivated varieties shapes the genetic structure of sweet chestnut (Castanea sativa Mill.) populations
Source: Sci Rep. 2022 Sep 2;12:15007. doi: 10.1038/s41598-022-17635-9 (PMC9440197; doi:10.1038/s41598-022-17635-9)
Supplement: Supplementary file 1 — Supplementary Information. [file 41598_2022_17635_MOESM1_ESM.pdf]

**Table S1.** Analysis of molecular variance (AMOVA).

| Source of variation | *df* | Variance components | % Total variation | *Fst* | *P* |
|---|---|---|---|---|---|
| Among populations | 2 | 0.450 | 10.83 | 0.108 | < 0.0001 |
| Within populations | 435 | 3.705 | 89.17 | | |

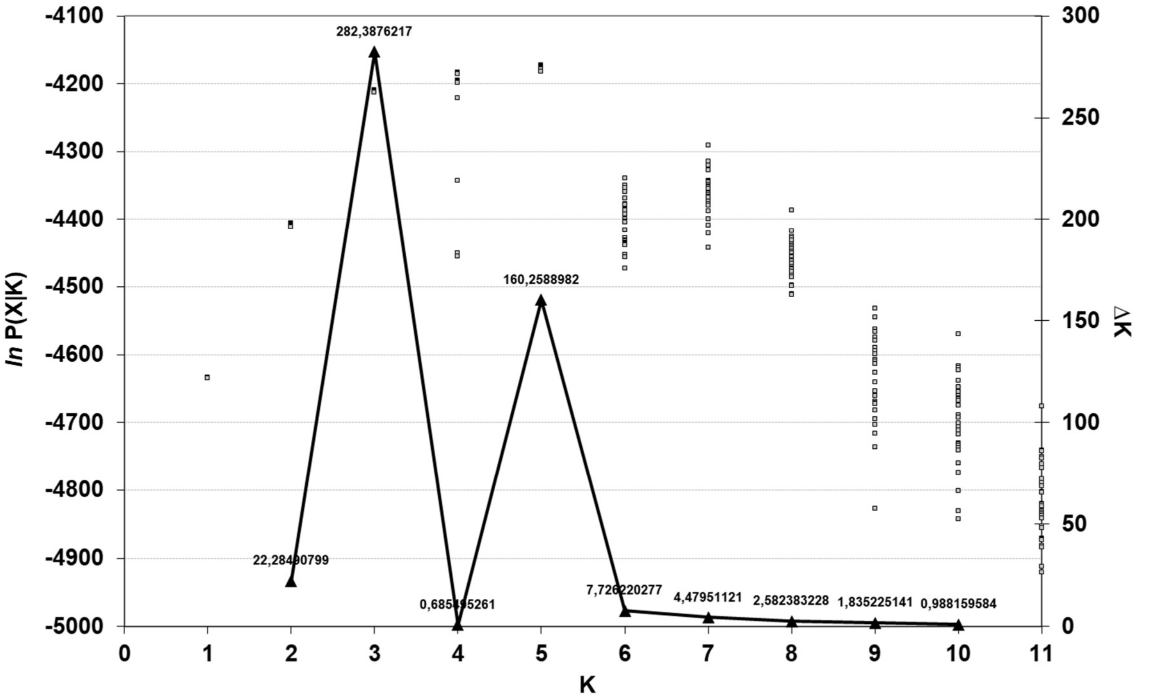


**Figure S1.** Inference of K, the most probable number of clusters, using STRUCTURE software, based on microsatellite analysis of 219 total samples of sweet chestnut.

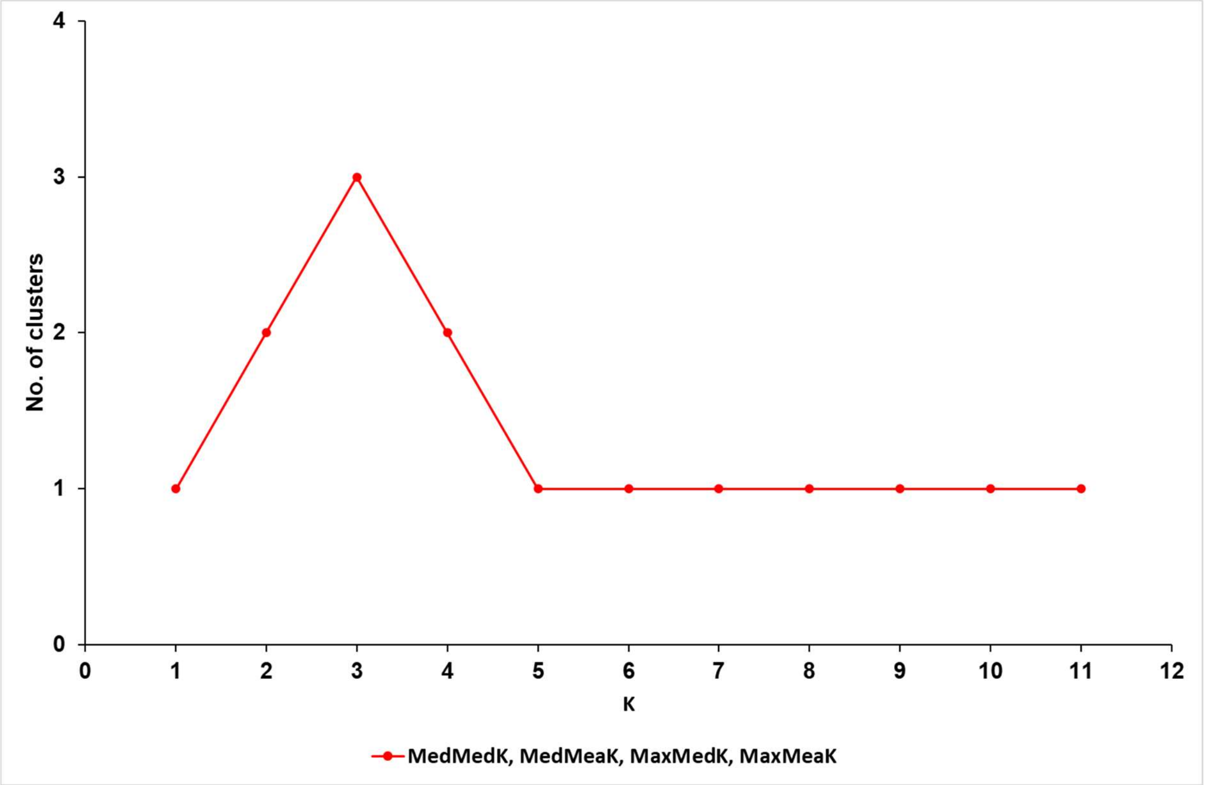


**Figure S2.** The optimal number of clusters calculated by MedMeaK, MaxMeaK, MedMedK and MaxMedK as implemented in StructureSelector.
